# Over-Expression of *Arabidopsis EDT1* Gene Confers Drought Tolerance in Alfalfa (*Medicago sativa* L.)

**DOI:** 10.3389/fpls.2017.02125

**Published:** 2017-12-13

**Authors:** Guangshun Zheng, Cunying Fan, Shaokang Di, Xuemin Wang, Chengbin Xiang, Yongzhen Pang

**Affiliations:** ^1^Key Laboratory of Plant Resources and Beijing Botanical Garden, Institute of Botany, Chinese Academy of Sciences, Beijing, China; ^2^Institute of Animal Science, Chinese Academy of Agricultural Sciences, Beijing, China; ^3^University of Chinese Academy of Sciences, Beijing, China; ^4^School of Life Sciences, University of Science and Technology of China, Hefei, China

**Keywords:** alfalfa, drought tolerance, *AtEDT1* gene, transgenic alfalfa, over-expression

## Abstract

Alfalfa (*Medicago sativa* L.) is an important legume forage crop with great economic value. However, as the growth of alfalfa is seriously affected by an inadequate supply of water, drought is probably the major abiotic environmental factor that most severely affects alfalfa production worldwide. In an effort to enhance alfalfa drought tolerance, we transformed the *Arabidopsis Enhanced Drought Tolerance 1* (*AtEDT1*) gene into alfalfa *via Agrobacterium*-mediated transformation. Compared with wild type plants, drought stress treatment resulted in higher survival rates and biomass, but reduced water loss rates in the transgenic plants. Furthermore, transgenic alfalfa plants had increased stomatal size, but reduced stomatal density, and these stomatal changes contributed greatly to reduced water loss from leaves. Importantly, transgenic alfalfa plants exhibited larger root systems with larger root lengths, root weight, and root diameters than wild type plants. The transgenic alfalfa plants had reduced membrane permeability and malondialdehyde content, but higher soluble sugar and proline content, higher superoxide dismutase activity, higher chlorophyll content, enhanced expression of drought-responsive genes, as compared with wild type plants. Notably, transgenic alfalfa plants grew better in a 2-year field trial and showed enhanced growth performance with increased biomass yield. All of our morphological, physiological, and molecular analyses demonstrated that the ectopic expression of *AtEDT1* improved growth and enhanced drought tolerance in alfalfa. Our study provides alfalfa germplasm for use in forage improvement programs, and may help to increase alfalfa production in arid lands.

## Introduction

Alfalfa (*Medicago sativa* L.) is a widely planted, perennial legume forage crop; its growth requires little or no nitrogen fertilizer, as alfalfa has the ability to fix nitrogen through symbiotic bacteria (Jiang et al., 2009; Duan et al., 2015). As a high-yielding and high-quality forage crop, alfalfa is extensively planted around the world. However, in many areas, water is a limiting factor for alfalfa yield, and drought seriously affects the growth and production of alfalfa worldwide. As a result, improving drought tolerance is a priority target trait in alfalfa breeding.

It is now well established that transcription factors (TFs) play important roles in signal transduction related to plant tolerance, from the perception of an environmental stress to late response mediated by downstream genes (Holmberg and Bulow, 1998). Both structural and TF genes are known to respond to drought stress via complex and orderly networks, ensuring normal plant growth and development. Among them, several different types of TFs have been shown, via ectopic expression, to be effective for increasing plant drought tolerance in a variety of plant species: these include DREBs, MYBs, bZIPs, bHLHs, and NACs TFs (Yamaguchi-Shinozaki and Shinozaki, 2006; Agarwal et al., 2017; Nakashima and Suenaga, 2017). For example, the *DREB2.7* gene from maize enhanced drought tolerance in transgenic *Arabidopsis* (Liu et al., 2013). *CmMYB2* from *Chrysanthemum* increased resistance against both drought and salinity stress in *Arabidopsis* (Shan et al., 2012). Both *CabZIP1* from pepper and *ZmbZIP72* from maize conferred drought and salt tolerance when expressed in *Arabidopsis* (Lee et al., 2006; Ying et al., 2012). Over-expression of *TabHLH39* from wheat significantly enhanced drought, salt, and freezing tolerance in *Arabidopsis* during the seedling stage (Zhai et al., 2016). The *NAC* type TFs *MlNAC9* from *Miscanthus*, *ZmNAC55* from maize, *GhNAC2* from cotton, and *OsNAC6* from rice enhanced drought tolerance when expressed in various other plant species (Gunapati et al., 2016; Mao et al., 2016; Zhao et al., 2016; Lee et al., 2017). The use of these TFs to improve quantitative traits like drought tolerance is ideal, because they can simultaneously regulate the expression of many genes involved in drought response.

Conventional breeding typically takes long time to develop new drought-resistant varieties, so genetic modification strategies base on TFs have been explored to increase drought resistance in alfalfa improvement programs. TF genes that have been tested in genetic modification efforts in alfalfa originate from various plant species, including the model legumes *Medicago truncatula* and soybean. An ethyl-responsive *MtWXP1* gene from *M. truncatula* was transformed into alfalfa under the control of the CaMV35S promoter. The drought resistance of these transgenic alfalfa plants was increased significantly, and this increase attributed to improved cuticular wax accumulation on leaves, reduced water loss, and enhanced photosynthesis (Zhang et al., 2005; Jiang et al., 2009). In another study, a Cys2/His2-type zinc-finger protein gene *GsZFP1* from *Glycine soja* was expressed in alfalfa: it improved drought resistance by enhancing the expression of several stress-responsive genes in alfalfa (Tang et al., 2013). Besides TF genes, other genes such as the *Orange* gene from sweet potato, the *CodA* gene from bacteria, the *NHX* and *VP1* genes from *Zygophyllum xanthoxylum*, and the *ALDH* and *ZFP* genes from *Cleistogenes songorica* have also been introduced into alfalfa in efforts to improve drought tolerance (Bao et al., 2009, 2016; Tang et al., 2013; Li et al., 2014; Duan et al., 2015; Wang et al., 2015; Zhang et al., 2016). Although the molecular mechanisms through which the ectopic expression of these various genes affect drought resistance may differ, each of them did improve drought resistance of transgenic alfalfa.

*Arabidopsis Enhanced Drought Tolerance 1* (*AtEDT1*) encodes a protein of the homodomain-leucine zipper TF family in *Arabidopsis* (Schrick et al., 2004). The dramatic role of *AtEDT1* in enhancing drought tolerance was identified via activation tagging and subsequent analyses in *Arabidopsis* and tobacco (Yu et al., 2008). The activation of *EDT1* expression in *Arabidopsis* and tobacco improved multiple characteristics related to drought tolerance, including reduced stomatal density, increased root system extent, increased levels of superoxide dismutase (SOD) activity and thus increased tolerance to oxidative stress (Yu et al., 2008). Expression of the *AtEDT1* gene has remarkably improved the drought tolerance of multiple plant species, including rice, tobacco, sweet potato, wheat, poplar, and cotton (Yu et al., 2008, 2013, 2016; Ruan et al., 2012; Zhu et al., 2015; Li et al., 2016). Meanwhile, such transformation causes no obvious adverse morphological changes or penalties on yield. These studies suggest that *AtEDT1* appears to be an ideal candidate gene for use in attempts to improve drought resistance in alfalfa.

In recent years, the demand for alfalfa has been growing in the animal husbandry industry, the alfalfa planting area has expanded greatly in northern China. However, water scarcity is one of the most severe threats to alfalfa production in the arid region of northern China. In this study, we over-expressed the *AtEDT1* gene in the perennial legume forage alfalfa, and examined its influence on drought tolerance in both laboratory and field trials. We found that transgenic alfalfa plants showed significantly improved drought tolerance and biomass yield. Our results suggested that *AtEDT1* can be potentially used to develop new alfalfa varieties with high biomass yield and improved drought tolerance.

## Materials and Methods

### Vector Construction and Plant Transformation

The binary expression vector PCB2004-*AtEDT1* (Supplementary Figure S1) was kindly provided by Professor Xiang Chengbin from the University of Science and Technology of China (Yu et al., 2008). This vector was introduced into *Agrobacterium tumefaciens* strain GV3101 for transformation in alfalfa.

Young leaves of alfalfa (Zhongmu No. 1) were used as explants for transformation via a previously described protocol (Cosson et al., 2015). Briefly, leaf disks were dipped into *Agrobacterium* cultures re-suspended in SH3a liquid medium for 30 min. After draining the excessive liquid, leaf disks were put on SH3a solid medium without any antibiotics for 2 days in the dark in a tissue culture room. Subsequently, leaf disks were moved to new SH3a medium containing antibiotics (200 mg/L cefotaxime and ticarcillin) and 3 mg/L phosphinothricin in darkness for about 5–6 weeks. During the process of regeneration, the calli were transferred to SH9 medium containing 150 mg/L kinetin and 3 mg/L phosphinothricin for the first 3 weeks. The calli were then transferred to new SH9 medium containing 150 mg/L kinetin and antibiotics (200 mg/L cefotaxime and ticarcillin) for seedling regeneration. Seedlings were further transferred to fresh SH9 medium containing 500 mg/L indole-3-acetic acid for rooting.

### Screening and Identification of Transgenic Lines by PCR

To screen transgenic lines, genomic DNA from young leaves of ppt-resistant lines were extracted by using a cetyltrimethyl ammonium bromide protocol (Permingeat et al., 1998). All regenerated alfalfa plants were confirmed by PCR with primers AtEDT1F and AtEDT1R (Supplementary Table S1), and the PCR procedure was as follows: denatured at 94°C for 8 min, followed by 35 cycles of 94°C for 30 s, 56°C for 45 s, and 72°C for 1 min, then extended at 72°C for 10 min.

The expression level of the *AtEDT1* gene in transgenic alfalfa plants were measured by RT-PCR. Total RNA was extracted from young leaves of PCR-positive lines and wild type plants using a TRIzol Kit (TianGen, China) according to the manufactures’ instruction. First-strand cDNA was synthesized by using a HiFiScript Quick gDNA Removal cDNA Kit (CWBiotech, China) according to the product manual. cDNA samples were amplified with primers AtEDT1F and AtEDT1R (Supplementary Table S1), the procedure was as followed: denatured at 94°C for 8 min, followed by 30 cycles of 94°C for 30 s, 56°C for 45 s, and 72°C for 1 min, then extended at 72°C for 10 min. *ACTIN2* gene was used as internal control with primers MsACTIN2F and MsACTIN2R (Supplementary Table S1). Both the PCR and RT-PCR products were separated on 1% agarose gels with ethidium bromide and visualized with an ultraviolet gel imaging system.

### Plant Materials and Stress Treatments

All transgenic and wild type alfalfa lines were propagated by cutting, and they were used for further culture and analysis when they were approximately 25 cm high. For the drought stress treatment, eight transgenic lines were transplanted into plastic culture pots (10 cm × 12 cm) containing 80 cm^3^ of vermiculite and nutrient soil (1:1) and grown under 16-h (day) photoperiod, a temperature of 24°C ± 3°C, and a relative humidity of 30%. Alfalfa plants were watered every 2 days for 3 weeks, then they were deprived of water for 20 days, followed by re-watering for another 5–10 days.

For treatment with PEG6000 (mock drought treatment), wild type and transgenic line OE-11 plants were watered with the same volume (100 mL) of 20% PEG6000, and leaves at the same position were collected at 0, 1, 3, 6, 12, and 24 h for RNA extraction and further gene expression analysis.

### Measurement of Water Content

Water content was measured as described by Yu et al. (2008) with some minor modifications. Young leaves (about 0.5 g) of transgenic and wild type plants grown under normal conditions were detached, and weighed immediately on weighing paper with an analytical balance. Then, these leaves were placed on a laboratory bench (26% relative humidity, 20°C) and weighed at designated time intervals (each 0.5 h) over a total period of 9 h. Three replicates were performed for each line. Relative water content was calculated against the initial weight of leaves, which were set as 100% for each plant at 0 h.

### Determination of the Size and Density of Stomatal and Epidermal Cells

Young leaves at similar positions were sampled from transgenic and wild type plants that were approximately 25 cm high. The leaf imprint method was used as previously described by Yu et al. (2008). Briefly, a drop of transparent nail polish was applied on the adaxial side of the sampled leaves, and the dried imprints were separated from the leaves about 5 min later. Leaf imprints were observed on glass slides under a stereo microscope. For statistical analysis, at least five leaves were sampled from each plant, with three plant samples for each line.

### Measurement of Relative Membrane Permeability and MDA Content

Membrane permeability can be inferred from the electrolyte leakage rate, which was measured with a conductivity meter according to Tang et al. (2013). Briefly, three leaf disks in leaf segments from three plants were vacuum-infiltrated in deionized water for 20 min and maintained in water for 2 h. The conductivities (C1) of the obtained solutions were then determined. Next, leaf segments in deionized water were boiled for 15 min. After being cooled to room temperature, the conductivities (C2) of the resulting solutions were determined again. The values of C1 to C2 (C1/C2) were calculated and used to evaluate relative electrolyte leakage.

Malondialdehyde (MDA) content was measured using a modified thiobarbituric acid method as described by Hodges et al. (1999). Approximately 0.3 g of leaf tissues were ground in 10 mL buffer containing 0.1 M K_2_HPO_4_-KH_2_PO_4_ (pH 7.6), 1 mM EDTA, 0.3% Triton X-100, 4% PVPP, using quartz sand with a mortar and pestle. The homogenate was centrifuged at 10,000 rpm for 20 min. The reaction mixture containing 200 μL of extract and 600 μL of 5% trichloroacetic acid, 200 μL 0.67% thiobarbituric acid was heated at 95°C for 30 min, quickly cooled on ice, and then centrifuged again at 10,000 rpm for 20 min. The absorbance at 450, 532, and 600 nm were measured using an ultraviolet spectrophotometer, *C* (μM) = 6.45 × (*A*_532_ -*A*_600_) - 0.56 × *A*_450_. Three biological replicates were performed.

### Measurement of Relative Total Soluble Sugar and Free Proline Content

Total soluble sugar content was assayed by using the anthrone method as described by Bailey (1958). Briefly, fresh alfalfa leaves (0.5 g) were cut into pieces, and then boiled in 15 mL deionized water for 20 min. After cooling to room temperature, they were filtered into a 100 mL conical flask, rinsed several times with deionized water to make 100 mL. Then 1.0 mL extraction was added to test tube with 5 mL anthrone, then boiled 10 min at 100°C. After cooling to room temperature, samples were analyzed with a spectrophotometer at 620 nm with water instead of extraction solution as the blank. The soluble sugar content *X* (μg/g FW) was quantified by the anthrone method using glucose as the standard. *C* (%) = *X* × *V*_t_ × *n* × 100 ÷ (*V*_1_ × FW × 10^6^), where *V*_t_ is the total extraction volume, *n* is the dilution multiple, *V*_1_ is the sample volume, and FW is the fresh weight.

Free proline content was measured as described by Bates et al. (1973) with minor modifications. Leaf tissue samples (0.5 g) were boiled in 3% sulfosalicylic acid for 10 min with constant shaking. After cooling and filtering, 2 mL of the filtrate was mixed with the same volume of glacial acetic acid, with acidic ninhydrin reagent as the visualization reagent. The mixtures were then analyzed at 520 nm with a spectrophotometer.

### Measurement of Relative SOD Activity, Total Chlorophyll Content, and Flavonoid Content

Superoxide dismutase activity was assayed by using the nitro blue tetrazolium (NBT) photocolorimetric method according to Zhang et al. (2014). SOD crude extractions were collected as described for the measurement of MDA content. And 3 mL reaction mixture was added to a test tube containing 1.5 mL of 50 mM K_2_HPO_4_-KH_2_PO_4_ (pH 7.6), 0.3 mL 130 mM methionine, 0.3 mL 750 μM NBT, 0.45 mL 20 μM lactoflavin, 0.3 mL 100 μM EDTA-Na_2_, 0.15 mL SOD extraction solution. They were then blended, and two test tubes were set as blanks with phosphate buffer solution instead of extraction solution (one was in the dark, while the other was placed under 4000 lx fluorescent lamp for 20–30 min at 25–35°C). They were measured with a spectrophotometer at 560 nm with a blank tube in the dark as the blank control. The SOD activity was calculated as SOD = (*A*_0_ - *A*_s_) × *V*_t_ ÷ (*A*_0_ × 0.5 × FW × *V*_1_), where *A*_0_ is the absorbance value of contrast tube in light, *A*_s_ is the absorbance value of sample tube, *V*_t_ is the total extraction liquid volume, *V*_1_ is sample volume, and FW is the fresh weight.

Total chlorophyll content was determined as described previously (Amid et al., 2012). About 0.5 g of leaf samples were used in the assay, which were immersed in a solution containing 30 mL 80% (V/V) ethanol. The mixtures were gently vibrated on a rotary shaker in the dark until the green pigmentation was absent from the leaves. The absorbance was measured at 654 nm (*A*_654_). Total chlorophyll content was calculated as: *C* (mg/g) = *A*_654_ × *V*_t_ ÷ (39.8 × FW), where *V*_t_ is the extraction volume (mL), and FW is the fresh weight.

Total flavonoids were extracted as described by Pang et al. (2007). And 400 μL water, 30 μL 5% NaNO_2_, 30 μL 10% AlCl_3_, 200 μL 1 M NaOH, and 240 μL water were added sequentially to 100 μL of the extract. The absorption of the final mixtures were measured at 510 nm with a spectrophotometer. Total flavonoid content was calculated with quercetin as the standard.

### Validation of Plant Growth and Photosynthesis in a Field Trial

A field trial was performed in an isolated plot from June 10, 2016 inside an Experimental Station of Beijing Botanical Garden located in semi-arid region. Wild type and three transgenic alfalfa plant lines (OE-4, OE-11, and OE-13) were vegetatively propagated by cutting and grown in a greenhouse for about 60 days. Then, 18 plants for each line (of similar height, about 5 cm) were planted in the isolated plot of about 60 m^2^ in area. After transplanting, all alfalfa plants were watered to ensure their survival, and then water application was ceased for the remaining 90 days of the trial. Growth performance was measured every 30 days for a total of 90 days, and the fresh weight of aerial and underground parts of all of the alfalfa plants were measured at 90 days. Photosynthetic rates were measured by using a portable photosynthesis system Li-6400 in the morning (9–10 am) at day 30, 60, and 90.

### Quantitative Real-Time PCR

Total RNA was extracted from leaves of eight transgenic lines, as well as transgenic line OE-11 and wild type alfalfa plants treated with 20% PEG6000 at 0, 1, 3, 6, 12, and 24 h, by using TRNzol-A^+^ Reagent (Tiangen Biotech, Beijing, China). One microgram of RNA was then used as the template for reverse transcription, using a HiFiScript gDNA Removal cDNA Synthesis Kit (CWBIO, Beijing, China). qRT-PCR was performed with UltraSYBR Mixture (CWBIO, Beijing, China) in a volume of 20 μL with 1 μL diluted cDNA (1:5). Primer sequences for drought-related genes and internal control gene are listed in Supplementary Table S1. Expression levels of all drought-responsive genes were determined by the 2^-ΔΔ*C_T_*^ method, with three biological replicates and three technical replicates. For the measurement of the *AtEDT1* in the eight transgenic lines, the expression level in line OE-8 was set as a value of 1. For the PEG6000 treatment, the expression levels of each gene at 0 h for the wild type were set as a value of 1.

### Statistical Analysis

Statistically significant differences (*P* < 0.05 or *P* < 0.01) were calculated based on Student’s *t*-tests. Data are presented as the means ± SD of 3, 6, or 18 independent replicates.

## Results

### Generation of Transgenic Alfalfa Plants Over-Expressing *AtEDT1*

**FIGURE 1:**
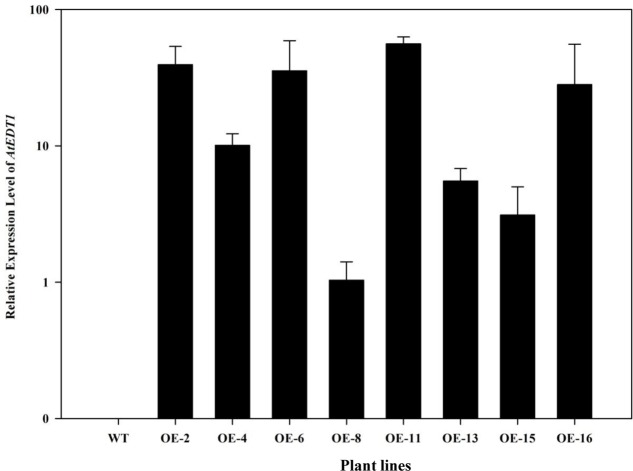
Expression levels of *AtEDT1* in wild type and transgenic alfalfa plants as revealed by qRT-PCR analysis. *ACTIN2* gene was used as house-keeping control. WT, wild type control plant; transgenic alfalfa line No. 2, 4, 6, 8, 11, 13, 15, and 16. Expression of *AtEDT1* in OE-8 with the relative low expression level was set as a value of 1.

To investigate the drought tolerance potential of *AtEDT1* in alfalfa, the open reading frame of *AtEDT1* was introduced into alfalfa via *Agrobacterium*-mediated transformation. Twenty-one independent phosphinothricin-resistant lines were produced and further confirmed by PCR analysis (Supplementary Figure S2). RT-PCR and qRT-PCR analysis revealed that *AtEDT1* was expressed at various levels, and eight lines with relatively high expression levels were selected for further analysis (**Figure 1**). These results indicated that *AtEDT1* was successfully introduced and expressed in alfalfa plants.

### *AtEDT1* Obviously Enhanced Drought Tolerance in Alfalfa

**FIGURE 2:**
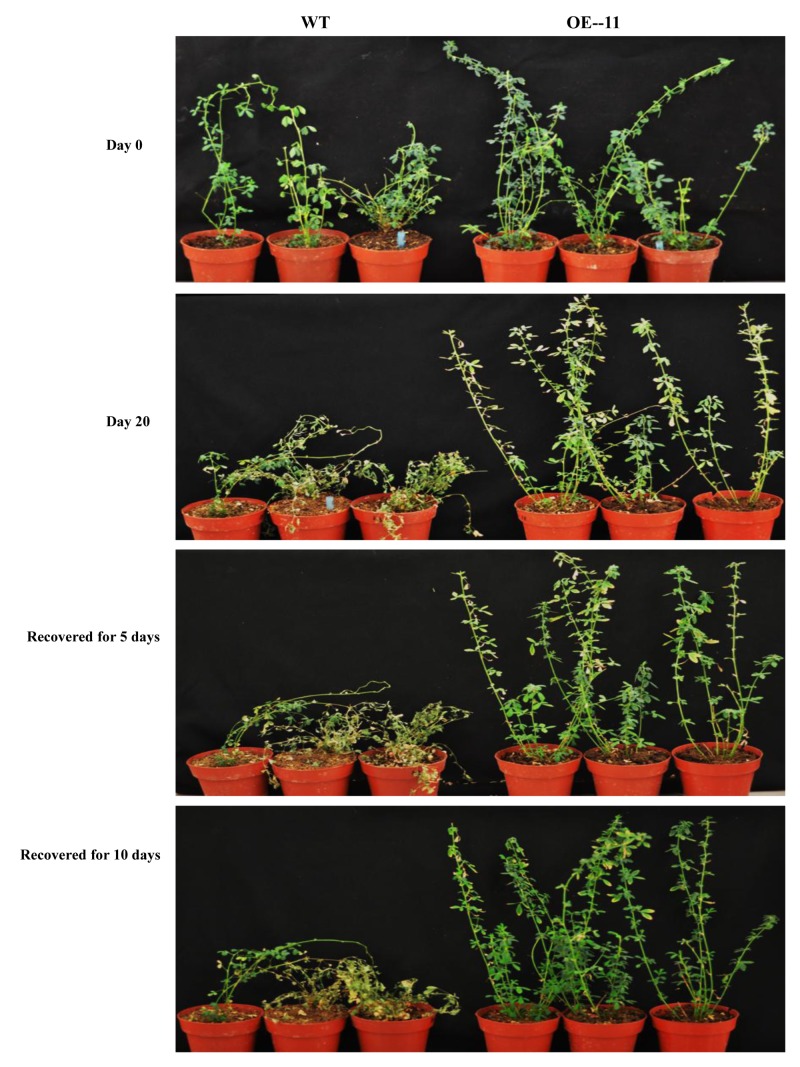
Growth performance of the *AtEDT1* transgenic alfalfa under normal and drought stress conditions. Day 0: wild type and transgenic alfalfa lines without any drought stress; Day 20: 20 days after drought stress treatment; Recovered for 5 days: recovered for 5 days after 20-day drought treatment; Recovered for 10 days: recovering for 10 days after 20-day drought treatment.

**FIGURE 3:**
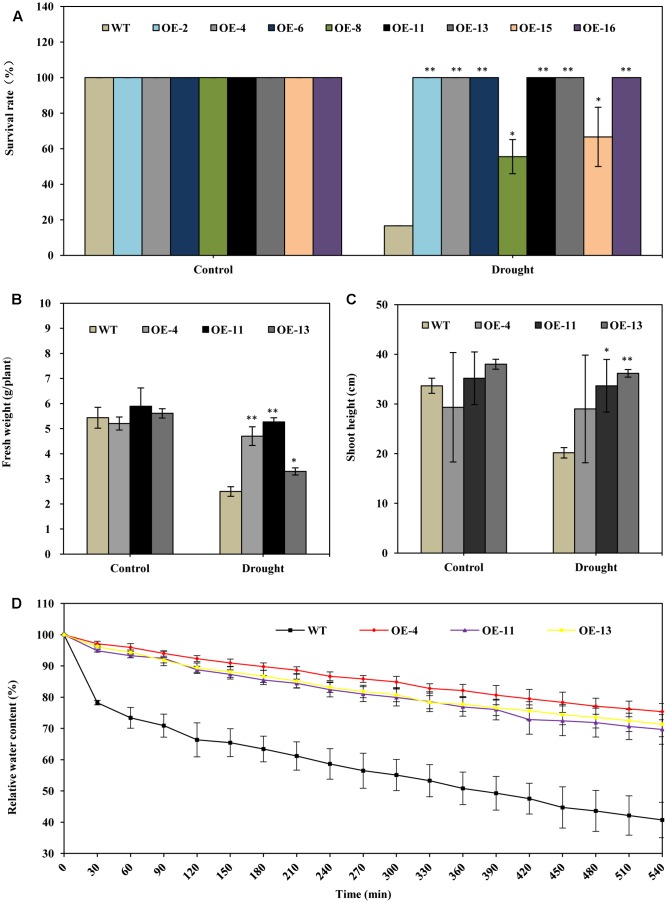
*AtEDT1* significantly enhanced the drought tolerance of transgenic alfalfa plants. **(A)** Survival rate of the alfalfa plants after drought treatment followed by 10-day-recovery. Values are means ± standard deviations (SD) of six replicates (^∗^*P* < 0.05, ^∗∗^*P* < 0.01). **(B,C)** Fresh weight and shoot height of alfalfa after 10-day-recovery. Values are means ± SD of six replicates (^∗^*P* < 0.05, ^∗∗^*P* < 0.01). **(D)** Water content of wild type and transgenic alfalfa plants. Detached leaves (around 0.5 g) were exposed to air, and fresh weight was measured at the indicated time points. Values are means ± SD of six replicates.

Under drought stress treatment, plants of all of the transgenic lines grew better and showed less wilting leaves than did wild type plants at 20 days after watering was ceased (**Figure 2**). When the plants were re-watered for 5 days, plants of all of the transgenic lines recovered more completely and more rapidly than did wild type plants (**Figure 2**). When the plants were re-watered for 10 days, most wild type plants failed to recover: only about 17% of wild type plants survived (**Figures 2**, **3A**). In contrast, most of the transgenic plants recovered to a normal state, and more than 67% survived (**Figures 2**, **3A**). From the eight transgenic lines, lines OE-4, OE-11, and OE-13, each of which had a survival rate of 100% were selected for further analysis (**Figure 3A**).

The fresh weight of transgenic lines OE-4, OE-11, and OE-13 were 88, 111, and 32% greater than wild type after drought treatment (**Figure 3B**). The shoot lengths of the transgenic plants were slightly longer than those of the wild type plants after drought treatment; no significant difference in root lengths between the wild type and transgenic plants were observed before drought treatment (**Figure 3C**).

We also analyzed the water content in both transgenic and wild type lines. It revealed that the water content of detached leaves from transgenic alfalfa plants were significantly higher than that of wild type under drought stress (**Figure 3D**). The relative water content in the three transgenic lines were higher than wild type, respectively, by 29–35% at 540 min (**Figure 3D**). This result indicated that over-expression of *AtEDT1* can reduce water loss in transgenic lines. Taken together, our results suggest that over-expression of *AtEDT1* can significantly improve the drought tolerance of alfalfa plants.

### *AtEDT1* Affects Leaf-Related Properties in Alfalfa

**FIGURE 4:**
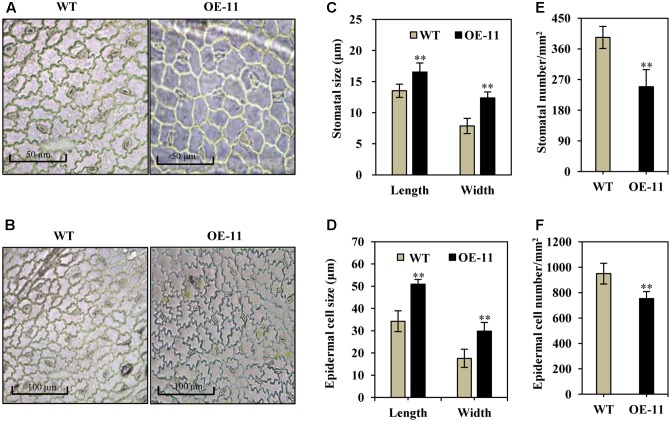
Enlarged stomata size, reduced stomatal and epidermal cell density in *AtEDT1* transgenic alfalfa plants. **(A,B)** Comparison of adaxial epidermal imprint images of wild type (left) and transgenic line OE-11 (right). A surface imprint was used as described in the Experimental Procedures. Bar = 50 μm **(A)**. Bar = 100 μm **(B)**. **(C)** Stomata size of wild type and transgenic line OE-11. One leaf from similar position of wild type and transgenic line OE-11 was respectively sampled for surface imprint. Values are means ± SD of 13 stomata (^∗∗^*P* < 0.01). **(D)** Stomatal cell density of wild type and transgenic line OE-11. Values are means ± SD of five images (^∗∗^*P* < 0.01). **(E)** Epidermal cell size of wild type and transgenic line OE-11. Values are means ± SD of 10 cells (^∗∗^*P* < 0.01). **(F)** Epidermal cell density of wild type and transgenic line OE-11. Values are means ± SD of four images (^∗∗^*P* < 0.01).

Stomatal size and density are known to be important for water and CO_2_ exchange; we thus measured stomata size and density in transgenic and wild type alfalfa plants. Under normal growth conditions, the stomata were often in an open state in wild type plants (**Figures 4A,B**, left), whereas most of the stomata were in a closed state in the transgenic line (OE-11, **Figures 4A,B**, right). Additionally, stomatal size increased significantly in the *AtEDT1* transgenic alfalfa plants as compared to wild type, in both length and width (29 and 57% greater than wild type, respectively) (**Figure 4C**). The average stomatal density of transgenic plants were reduced by 37% as compared to wild type plants (**Figure 4D**).

Epidermal cell size increased significantly in *AtEDT1* transgenic plants as compared to wild type plants (**Figures 4A,B**), in both length and width (49 and 69% greater than wild type, respectively) (**Figure 4E**). The average epidermal cell density of the transgenic line was decreased by 21% compared to the wild type plants (**Figure 4F**). These results together suggest that greater stomatal cell size and reduced stomatal cell density contribute to the observed reduction in water loss, thereby confer improved drought tolerance in the *AtEDT1* transgenic alfalfa plants.

### *AtEDT1* Enlarged the Root System in Transgenic Alfalfa Plants

**FIGURE 5:**
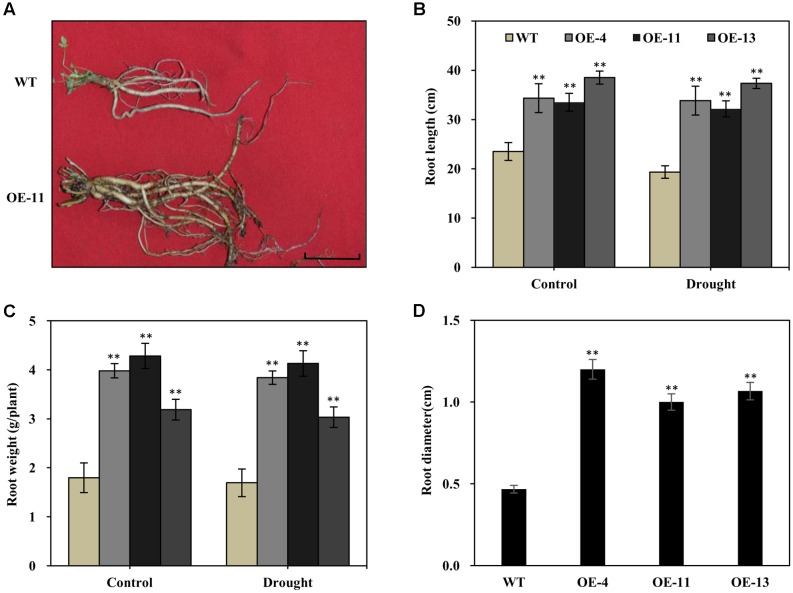
Increased root length, weight, diameter, and chlorophyll content in the *AtEDT1* transgenic alfalfa plants. **(A)** Root phenotype of wild type and transgenic alfalfa plants. Wild type and transgenic alfalfa plants with or without drought stress treatment were carefully dug out, and washed thoroughly with water. Bar = 5 cm. **(B–D)** Root length and fresh weight of wild type and transgenic alfalfa plants after drought stress treatment. Values are means ± SD of three replicates (^∗^*P* < 0.05, ^∗∗^*P* < 0.01). **(D)** Root diameter of wild type and transgenic alfalfa plants before drought stress treatment. Values are means ± SD of three replicates (^∗^*P* < 0.05, ^∗∗^*P* < 0.01).

It is known that alfalfa plants with longer and thicker root systems can gain more water and nutrients in environments experiencing drought. We thus examined the root morphology of *AtEDT1* transgenic alfalfa plants in comparison with wild type under normal and drought conditions. Under normal conditions, roots of transgenic plants from OE-4, OE-11, and OE-13 were 46, 43, and 64% longer, respectively, than those of wild type plants (**Figures 5A,B**). After 20 days of drought stress treatment, roots of both the transgenic and the wild type plants were longer, but the root lengths of transgenic plants were significantly longer than those of wild type plants (**Figure 5B**).

Similar to root length, fresh root weight was significantly greater in the transgenic plants than in wild type plants, in both the normal and the drought stress condition (**Figure 5C**). After 20 days of drought stress, the fresh root weight of plants of the OE-11 transgenic line was 1.4-fold greater than that of wild type plants (**Figure 5C**).

Under normal growth condition, the transgenic plants had thicker roots than wild type plants. The root diameters of plants of transgenic lines OE-4, OE-11, and OE-13 were 1.6-, 1.1-, and 1.3-fold thicker than that of wild type plants, respectively (**Figure 5D**).

### Expression of *AtEDT1* Leads to Changes in Physiology Parameters in Transgenic Alfalfa Plants

Cell membranes play important roles in plant stress responses, and cell membrane permeability typically increases upon stress. Increased cell membrane permeability raises the extent of cellular damage, negatively contributing to stress tolerance. To determine possible changes in membrane permeability under drought stress, membrane permeability was measured in both transgenic lines and wild type plants.

**FIGURE 6:**
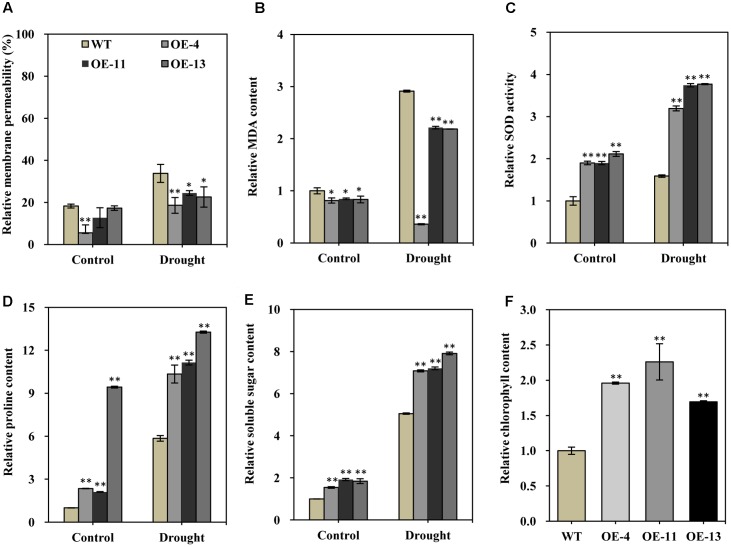
Relative membrane permeability, MDA content, SOD activity, soluble sugar content, proline content, and chlorophyll contents of wild type and transgenic alfalfa plants before and after drought stress treatment. Comparison of leaf membrane permeability **(A)**, MDA content **(B)**, SOD activity **(C)**, proline content **(D)**, soluble sugar content **(E)**, and relative chlorophyll content **(F)** of wild type and transgenic alfalfa plants before and after 20-day drought treatments. Values are means ± SD of three replicates (^∗^*P* < 0.05, ^∗∗^*P* < 0.01).

Under normal growth conditions, the relative membrane permeability was lower in the transgenic plants than in wild type plants (**Figure 6A**). Upon drought treatment, the relative membrane permeability of both transgenic plants and wild type plants were increased (**Figure 6A**). However, the increase in the relative membrane permeability of the transgenic plants was obviously less pronounced than in the wild type (**Figure 6A**). MDA is a product of lipid peroxidation, which is an index of the generation of toxic oxygen species. Similar to relative membrane permeability, MDA also negatively contributes to stress tolerance. We found that the relative MDA content was markedly lower in transgenic plants than in wild type plants, in both normal growth conditions and drought conditions (**Figure 6B**), indicating that transgenic alfalfa plants suffered less severe oxidative damage than did wild type plants.

Superoxide dismutase can scavenge superoxide radicals under stress conditions, so higher SOD activity can positively improve stress tolerance. We found that SOD activity was significantly higher in transgenic plants than in wild type plants in both normal and drought conditions (**Figure 6C**). This result indicated that the transgenic plants had an enhanced capability to scavenge reactive oxygen species (ROS) as compared to the wild type.

Soluble sugars and proline are two common and important compatible osmolytes in higher plants, and they are thought to be involved in stress resistance. We therefore measured the content of both of these compounds before and after drought stress. This analysis revealed that both proline and soluble sugar content were obviously higher in the transgenic plants than in wild type plants under normal growth condition (**Figures 6D,E**). In particular, the proline content was more than 9.4-fold higher in plants of OE-13 than in wild type plants (**Figure 6D**). After drought stress, significantly increased proline and soluble sugar content was observed in both the transgenic plants and wild type plants (**Figures 6D,E**), but their content was significantly higher in the transgenic plants than in the wild type plants (**Figures 6D,E**). Therefore, higher soluble sugar and proline content appears to have positively contributed to the observed increased drought tolerance of the transgenic plants.

Photosynthesis is strongly affected by various stresses, and chlorophyll content is an indicator of photosynthesis activity; therefore, we also measured total chlorophyll content of alfalfa plants under normal conditions. About 96, 126, and 69% higher chlorophyll contents, respectively, were observed in transgenic plants as compared to wild type plants (**Figure 6F**). The significantly higher chlorophyll content may facilitate root growth in the transgenic lines by helping to maintain normal photosynthesis activity.

The total flavonoid content was increased in both transgenic and wild type plants under drought conditions compared to normal growth conditions (Supplementary Figure S3), although there was no significant difference between transgenic and wild type plants (Supplementary Figure S3). This result indicated that flavonoid compounds are inducible under drought stress, but were not affected by *AtEDT1* over-expression.

Considered together, our results indicate that over-expression of *AtEDT1* affects the accumulation of stress-associated compounds like soluble sugars, proline, MDA, and chlorophyll, and also affects the activity of stress-associated enzymes, like SOD.

### *AtEDT1* Affected the Expression of Drought-Responsive Genes in Transgenic Alfalfa Plants

**FIGURE 7:**
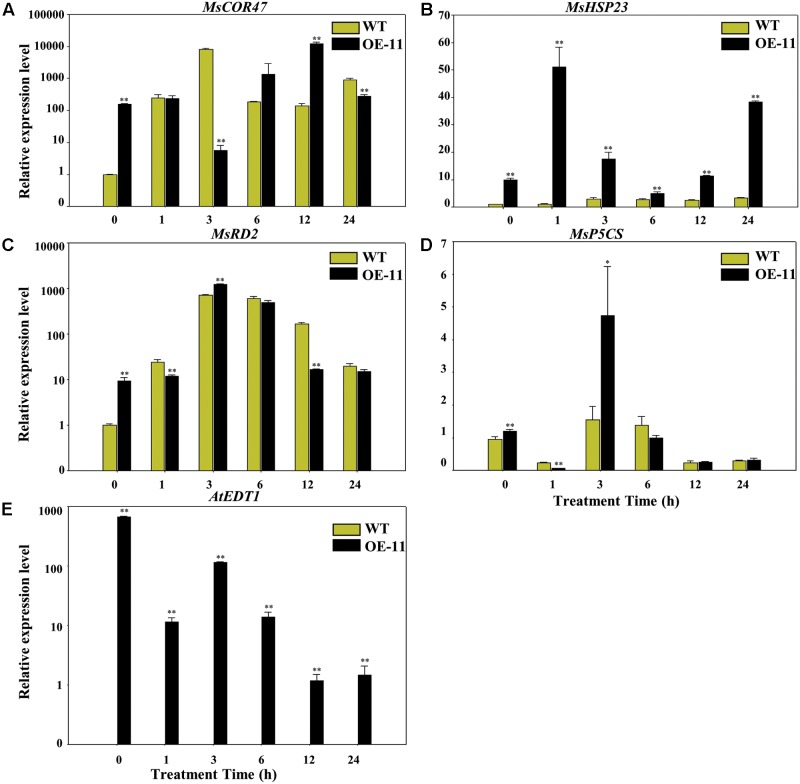
Relative expression levels of drought-responsive genes in wild type and transgenic alfalfa as detected by qRT-PCR. Relative expression levels of *MsCOR47* **(A)**, *MsHSP23* **(B)**, *MsRD2* **(C)**, *MsP5CS* **(D)**, and *AtEDT1* **(E)** genes in wild type and transgenic alfalfa plants treated with 20% PEG6000 at time points of 0, 1, 3, 6, 12, and 24 h. Values are means ± SD of three replicates (^∗^*P* < 0.05, ^∗∗^*P* < 0.01).

To gain more insight into the molecular mechanism of *AtEDT1* on alfalfa under drought stress, we analyzed the expression levels of *MsP5CS*, *MsHSP23*, *MsRD2*, and *MsCOR47* by qRT-PCR analysis. *MsP5CS* encodes a key enzyme in proline biosynthesis, and *MsHSP23*, *MsRD2*, and *MsCOR47* are drought-responsive genes that were investigated in previous studies in alfalfa and/or *Arabidopsis* (Huang et al., 2008; Tang et al., 2013; Quan et al., 2015). qRT-PCR analysis showed that the expression levels of these four genes were significantly higher in transgenic plants than in wild type plants under normal growth conditions at 0 h (**Figures 7A–D**). However, only the expression level of *MsHSP23* showed a constant increase in transgenic plants over the entire drought treatment period (**Figure 7B**). The expression level of *MsCOR47* was only higher at 6 and 12 h in transgenic plants compared to wild type plants, while the expression levels of both *MsRD2* and *MsP5CS* were only higher in transgenic plants than in the wild type at 3 h (**Figures 7A,C,D**), revealing that these genes respond variously under drought treatment in alfalfa. *AtEDT1* expression was detected in transgenic plants but not in wild type plants; its expression was decreased slightly under drought treatment (**Figure 7E**). Taken together, our results demonstrate that *AtEDT1* increased the expression of multiple drought-responsive genes, indicating a correlation between their expression level and drought tolerance in alfalfa.

### *AtEDT1* Improved Growth and the Photosynthetic Rate of Transgenic Alfalfa Plants under Field Conditions

**FIGURE 8:**
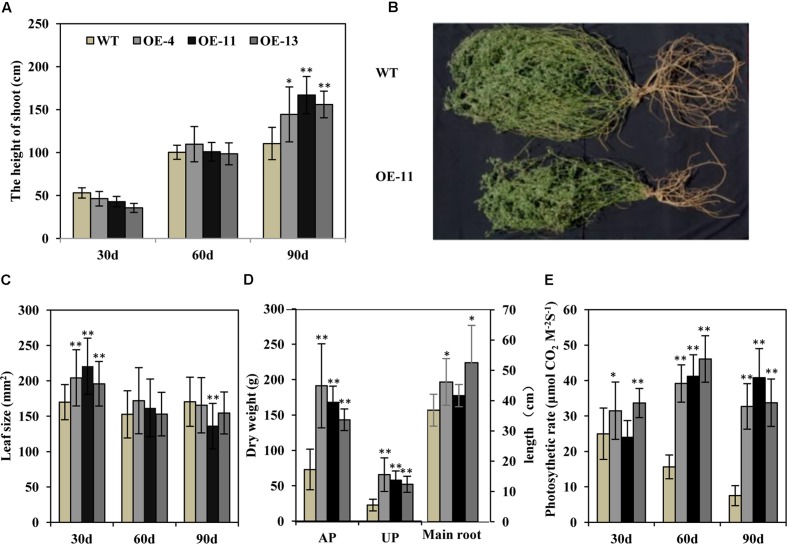
Effects of drought stress on wild type and transgenic alfalfa plants in a field trial. **(A)** Shoot height of wild type and transgenic plants at 30-, 60-, and 90-day in field trial. **(B)** Phenotype of wild type (upper panel) and transgenic (lower panel) plants after 90-day in field trial. **(C)** Leaf size of wild type and transgenic plants at 30-, 60-, and 90-day field test. **(D)** Left: dry weight of above-ground part (AP) and underground part (UP) in wild type and transgenic alfalfa plants. Right: length of main roots in wild type and transgenic alfalfa plants. **(E)** Photosynthetic rate of wild type and transgenic plants at 30-, 60-, and 90-day field trial. Values are means ± SD of three replicates (^∗^*P* < 0.05, ^∗∗^*P* < 0.01).

Transgenic alfalfa plants were planted in isolated plot in a semi-arid environment. Thirty and 60 days post-transplanting, the heights of the transgenic alfalfa plants were similar to wild type plants (**Figure 8A**). But after 90 days, the height of the plants of transgenic lines OE-4, OE-11, and OE-13 were, respectively, 1.3-, 1.5-, and 1.4-fold higher than the wild type plants (**Figure 8A**). These data suggest that the growth performance of the transgenic alfalfa plants was better than the wild type plants. After 90 days in the field, the transgenic plants displayed larger shoots and root systems than wild type plants (**Figure 8B**), but the leaf size of both the transgenic and wild type plans did not show any obvious difference within 90 days (**Figure 8C**). The biomass of the transgenic alfalfa plants were 1.9- to 2.9-fold greater than the wild type plants, whether for aerial shoot part or for underground root part (**Figure 8D**). Meanwhile, the main root length of plants of transgenic lines OE-4, OE-11, and OE-13 were, respectively, 25, 13, and 43% longer than that of wild type plants (**Figure 8D**).

We also measured photosynthetic rate and found that, during the first 30 days, the photosynthetic rates of the plants of transgenic lines OE-4 and OE-13 were higher than the wild type plants (**Figure 8E**). At 60 and 90 days, the photosynthetic rates of wild type plants decreased greatly due to drought stress, whereas those of transgenic plants were remarkably higher than wild type plants (**Figure 8E**).

**FIGURE 9:**
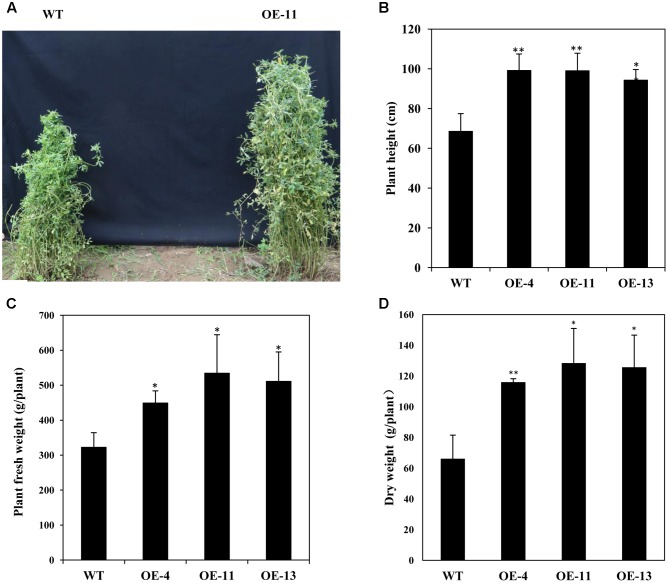
Growth of wild type and transgenic alfalfa plants in a field trial during the second year. **(A)** Phenotype comparison of wild type (left) and transgenic (right) plants in field trial during the second year. **(B)** Shoot height of wild type and transgenic plants at second year in field trial. **(C,D)** Fresh weight (left) and dry weight (right) of wild type and transgenic alfalfa plants. Values are means ± SD of three replicates (^∗^*P* < 0.05, ^∗∗^*P* < 0.01).

In addition, we measured the growth performance of alfalfa plant during June 2017 (in the second year of the field trial). We found that transgenic plants grew better than wild type plants with significant higher plant heights (**Figures 9A,B**), and remarkably increased plant fresh and dry weights (**Figures 9C,D**). Our results demonstrate that *AtEDT1* over-expression in alfalfa plants enhances drought tolerance and confer better growth performance under field conditions, which is most likely due to their relatively more extensive root systems and higher photosynthetic rates compared to wild type plants.

## Discussion

Alfalfa is the most widely grown legume forage crop worldwide, and it contributes greatly to global dairy, beef, and wool production. Improvement of drought tolerance through genetically modification is very important in alfalfa to face the challenge of water scarcity worldwide (Zhang et al., 2005).

### Over-Expression of *AtEDT1* Affects the Morphology and Physiology of Transgenic Alfalfa Plants under Drought Conditions

The *AtEDT1* gene can enhance the drought tolerance of alfalfa by changing the morphology of both aerial and underground part of transgenic alfalfa plants under drought conditions. Although no obvious morphology differences were observed between transgenic plants and wild type plants under normal growth conditions (**Figure 2**), transgenic alfalfa plants survived and recovered quickly after drought stress treatment, and displayed better growth performance than did wild type plants, in both laboratory and field tests (**Figures 2**, **8**).

On one hand, a better water status or health in transgenic plants was due to less water loss. Under severe water-deficient conditions, the survival of a plant will depend largely on its ability to restrict water loss through leaf epidermis (Rawson and Clarke, 1988). In our work, the leaf water content of all of the alfalfa plants decreased gradually under drought stress, although the water loss rate of the transgenic alfalfa plants was much lower than that of the wild type, suggesting that *AtEDT1* improve the water retention. These results were associated with reduced stomatal density, larger leaf stomatal size in the transgenic alfalfa plants (**Figure 4**), which decreased water transpiration and maintained leaf water content. Similar changes in stomata size and density were also found in cotton, poplar, tobacco, and pepper plants that were transgenically expressing *AtEDT1* (Yu et al., 2008, 2016; Zhu et al., 2015).

Meanwhile, less water loss in transgenic plants could also be explained by the increased soluble sugar and proline contents. High concentrations of soluble sugars can maintain a certain osmotic pressure, and high concentration of proline can stabilize the subcellular structure, reduce cell membrane permeability and improve free radical scavenging capability, except to function as osmotican (Li et al., 2016). These could help transgenic alfalfa plants maintain water content under low water potential conditions, and could help to promote the growth of transgenic plants under drought stress conditions.

On the other hand, a better water status or health in transgenic plants was due to greater water uptake. One would expect that a stronger root system should be one of the most important factors that contribute to the improved growth and enhanced drought tolerance of *AtEDT1* transgenic alfalfa. We found the length, weight and diameter of transgenic alfalfa roots were increased compared to the wild type, in both laboratory and field tests (**Figures 5**, **8**). Therefore, it appears that expression of *AtEDT1* confers drought tolerance by maximizing water uptake via production of a deeper and more substantial root system. The enlarged root system may be caused by the up-regulation of cell-wall-loosening proteins as a result of *AtEDT1* over-expression, as described in *Arabidopsis* (Xu et al., 2014). Meanwhile, enhanced JA biosynthesis might be another mechanism underlying the altered root architecture of alfalfa as reported in *Arabidopsis* (Cai et al., 2015). The root morphology changes that we observed in the present study are similar to those observed in rice, pepper, cotton, and poplar plants that were over-expressing *AtEDT1* (Yu et al., 2013, 2016; Zhu et al., 2015). Our study demonstrates that the over-expression of *AtEDT1* in alfalfa affects leaf-related morphology in a way that minimizes water loss, and affects root-related morphology to maximize water uptake and thereby further confer drought tolerance in transgenic alfalfa plants.

### *AtEDT1* Enhanced Oxidative Stress Response and Affected Gene Expression under Drought Conditions in Alfalfa

It is known that environmental stresses can cause the accumulation of harmful ROS substances that can damage cell membranes, DNA, and proteins (Apse and Blumwald, 2002); and timely removal of these substances is conducive to improvement of plant growth. In order to cope with this challenge, plants can produce lower MDA and ROS, thus to lower membrane permeability (Ruan et al., 2012). In our results, SOD activity was increased and MDA content was decreased in transgenic alfalfa plants, which suggested that over-expressing *AtEDT1* could enhance capability to deal with oxidative damage to cell membrane and improve drought tolerance.

The expression levels of *MsCOR47*, *MsHSP23*, *MsRD2*, and *MsP5CS* was increased in the transgenic alfalfa plants compared to the wild type plants under normal conditions (**Figure 7**). Further, under drought stress conditions, these genes responded differently, especially *MsHSP23*, which responded more significantly than other genes (**Figure 7**), suggesting *MsHSP23* may play important role in drought tolerance. The increased expression levels of these drought-responsive genes were also found in alfalfa over-expressing other TFs, as well as other plants species over-expressing *AtEDT1* under drought stress (Yu et al., 2008; Shan et al., 2012; Tang et al., 2013). Whether or not any other genes associated with growth performance and root architecture were affected by *AtEDT1* requires further investigation.

### *AtEDT1* Increased Alfalfa Biomass under Laboratory and Field Conditions

Plant yield is highly correlated with biotic and abiotic stresses, and drought is a major limiting factor for plant growth (Rockstrom and Falkenmark, 2000). Previous studies have demonstrated that over-expression of *AtEDT1* in rice increases biomass and kernel yield under both normal and drought conditions (Yu et al., 2013), and transgenic pepper over-expressing *AtEDT1* also showed better fruit yield (Zhu et al., 2015). In our study, *AtEDT1* transgenic alfalfa showed better performance and higher biomass yield under drought stress in laboratory conditions, which is different from *WXP1* transgenic alfalfa that showed moderately slow growth (Zhang et al., 2005). In particular, the photosynthetic rate of the *AtEDT1* transgenic alfalfa plants was higher as compared to wild type plants in field tests as in laboratory (**Figure 8**), which appears to have resulted in increased biomass in the transgenic plants, and the healthy status may indirectly contribute to the higher photosynthesis.

Genes ectopically over-expressed in alfalfa can improve transgenic plant drought tolerance under laboratory conditions (Zhang et al., 2005, 2016; Jiang et al., 2009; Tang et al., 2013; Li et al., 2014; Duan et al., 2015; Wang et al., 2015). However, very few transgenic alfalfa plants have been tested in field trials (Bao et al., 2016). In our study, expression of *AtEDT1* enhanced drought tolerance in alfalfa without a biomass yield penalty in a 2-year field trial. Therefore, *AtEDT1* transgenic alfalfa plants are promising germplasm for use in the breeding and development of alfalfa with enhanced drought tolerance and improved biomass yield.

## Conclusion

We generated transgenic alfalfa plants over-expressing *EDT1* from *Arabidopsis*, which showed better growth performance and enhanced drought tolerance under both laboratory and field conditions. Our results support previous conclusions that *AtEDT1* plays important roles in conferring drought tolerance in foreign plant species. This work suggests an approach for the production of alfalfa in arid and semi-arid regions of northern China, which could greatly benefit the animal husbandry.

## Author Contributions

YP, CX, and XW conceived and designed the experiment. GZ, CF, and SD performed the experiments. GZ, CF, SD, and YP analyzed the data. XW, CX, and YP contributed regents/material/analysis tools. YP and GZ wrote the paper.

## Conflict of Interest Statement

The authors declare that the research was conducted in the absence of any commercial or financial relationships that could be construed as a potential conflict of interest.
