# Supplementary material for: Over-Expression of Arabidopsis EDT1 Gene Confers Drought Tolerance in Alfalfa (Medicago sativa L.)
Source: Front Plant Sci. 2017 Dec 13;8:2125. doi: 10.3389/fpls.2017.02125 (PMC5733502; doi:10.3389/fpls.2017.02125)
Supplement: Supplementary file 1 [file Image_1.PDF]

Table S1 Primer sequences used in the present study

Gene name	Direction	Primer sequence (5'-3')
AtEDT1	Forward	5'-ATGAGTTTCGTCGTCGGCGT-3'
	Reverse	5'-GTCGTAAGAAGCCTGAGCAAT-3'
AtEDT1-RT	Forward	5'-AGCAGCATTGAACATCGCAA-3'
	Reverse	5'-TTGCCGTCGGTAAATTGCTT-3'
MsACTIN2	Forward	5'-TCAATGTGCCTGCCATGTATGT-3'
	Reverse	5'-ACTCACACCGTCACCAGAATCC -3'
MsRD2	Forward	5'-GCAGCTGTGGTTCTGGGGACC-3'
	Reverse	5'-AGCAATACTCACCGACGCTTCCT-3'
MsP5CS	Forward	5'-ATGGCGAACGCCGACCCTTGT-3'
	Reverse	5'-CGGCAACAGCCATCTCGCGT-3'
MsCOR47	Forward	5'-CGTTGCTTACGGTGGCGGTGC-3'
	Reverse	5'-TCCGGGTGGTGGTTCGGTGG-3'
MsHSP23	Forward	5'-CATTCAACACCAACGCCATG-3'
	Reverse	5'-CGGATCAAACACATCTGAGAGG-3'

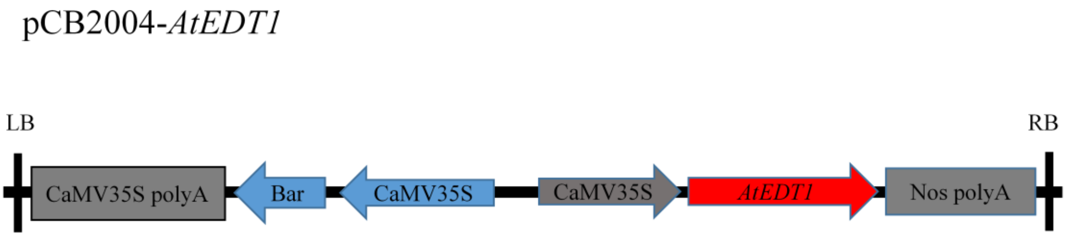

Figure S1. Schematic representation of the T-DNA region of binary vector pCB2004-AtEDT1 used in this study.

LB: left border, RB: right border, Bar: phosphinotricin acetyltransferase, CaMV35S: Cauliflower mosaic virus 35S promoter, Nos polyA: 3`-termination signal of nopaline synthase

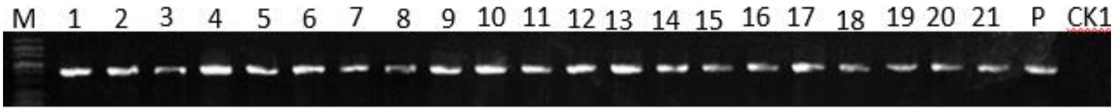

Figure S2. Screening of the transgenic alfalfa plants by PCR. M, DNA molecular weight marker; 1-21, transgenic lines; P, plasmid carrying *AtEDT1* as positive control; CK1, wild-type control.

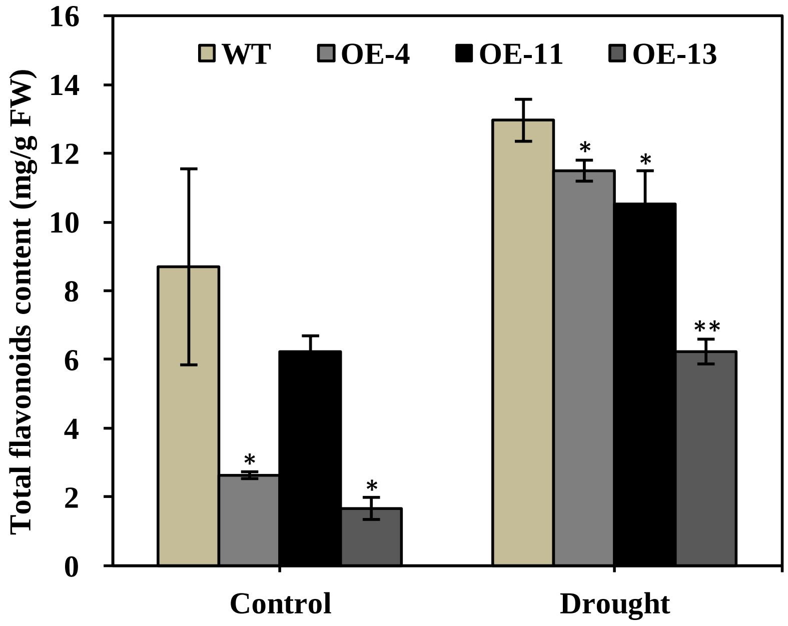

Figure S3. Total flavonoid content in wild-type and transgenic alfalfa plants with and without drought stress treatment.

Total flavonoid content from wild-type control and transgenic alfalfa plants with and without 20-day-drought stress treatment. Values are means ± SD of three replicates (*P < 0.05, **P < 0.01).
